# Supplementary material for: A Novel GPPAS Model: Guiding the Implementation of Antimicrobial Stewardship in Primary Care Utilising Collaboration between General Practitioners and Community Pharmacists
Source: Antibiotics (Basel). 2022 Aug 27;11(9):1158. doi: 10.3390/antibiotics11091158 (PMC9495087; doi:10.3390/antibiotics11091158)
Supplement: Supplementary file 1 [file antibiotics-11-01158-s001.zip › Supplementary_Table.pdf]

Supplementary Table S1: **International evidence and explanation in support of GPPAS model components**

GPPAS Model Component	International evidence and explanation in support of model components	Ref
Pharmacist-patient	A randomised controlled trial (RCT) study demonstrated that the use of patient-facing resources (e.g., take-home leaflets for patients) during CPs' routine consultation with a patient facilitated shared decision about the use of antimicrobial(s) [16]. This strategy was associated with an increased self-care advice and decreased patient referrals to GPs. Along with a CP in charge, other pharmacy staffs such as pharmacy assistants and technicians might advise patients or clients about the symptomatic treatment options and the usual duration of illness to manage self-limiting infection(s) such as viral upper respiratory tract infections (URTIs) or mild urinary tract infections (UTIs) without antimicrobial(s). The provision of advising patients that-"antimicrobials don't reduce the severity and duration of symptoms" and "the symptomatic relief is the best option to help recover the condition"- might bring benefits in improving patients' antibiotic seeking behaviour for treating minor infections [35].<br><br>In a German campaign, the improved communication with patients was successful in reducing 60% of antimicrobial consumptions [36]. There is evidence that patients who receive counseling from a CP demonstrate a better knowledge of the prudent use of antimicrobials [37]. This evidence underscores the importance of patient counselling by CPs on every antimicrobial prescription dispensed.	16, 35-37
GP-patient	According to Altiner et al [38], overprescribing of antibiotics by GPs might not be due to a lack of knowledge but a patient-centredness approach. In an Australian study, parents stated that they would visit multiple GPs if they believed that antibiotic was required for their child [39]. Surveyed GPs also expressed their concern about patient demands on antibiotics. This indicates that antibiotic prescriptions can be avoided in many cases if GPs can identify the concern and real expectations (e.g., symptoms relief, worry about the severity of disease) of the clients or patients. Therefore, a strategic guideline that outlines how to empower patients in therapeutic decisions for treating infection and communication skill training [40] would be beneficial and their effectiveness has been reported in the literature [17, 41].	17, 38-41
GP-pharmacist audit-feedback model	Such a model [47] led by an ambulatory care pharmacist demonstrated effectiveness in improving antibiotic prescribing for URTIs and UTIs	47-48

	in the United Sates: quality improvements were found in decision making when antibiotic is indicated, guideline-adherent choice and duration of antibiotic therapy [47].<br><br>Another study [48] showed that this model when led by an AMS physician and an AMS pharmacist together was highly effective to significantly improve guideline-concordant antibiotic prescribing from 38.9% to 57.9% in a family medicine clinic. Authors [48] also reported significant improvements in the selection (68.9% to 80.2%), dose (76.7% to 86.2%), and duration of antibiotic therapy (73.3% to 86.2%) according to antibiotic guideline.	
AMS education model	Van Katwyk et al [52] identified 94 AMS related educational programs globally in a systematic review with major gaps in the provision of these programs as accredited training program. The sustained impact is desirable if those programs were incorporated and regulated into the graduate curriculum of GPs and CPs [53].<br>In the UK, a GP-pharmacist consensus-based national AMS competencies curriculum has been developed for undergraduate GP and pharmacy professionals [53].	52-53
**GP-pharmacist interpersonal AMS education model**	Such a model in Scotland was effective to reduce the use of broad-spectrum antimicrobials in a large region as part of a national initiative [54]. A system supported GP-CP collaborative pharmacotherapy audit meetings model demonstrated the improvement in antibiotic prescribing in a randomised controlled trial [55]. A multimodal AMS education program implemented by a primary care team involving GPs and pharmacists showed a long-term impact on the sustained reduction of antibiotic prescribing and infections caused by *E. coli* in the community [56].<br>In the UK, a GP-pharmacist consensus-based national AMS competencies curriculum has been developed for undergraduate GP and pharmacy professionals [53]. Incorporation of shared case-based AMS learning modules into respective GP and pharmacy curricula may help educate future generation GPs and CPs about the importance of interprofessional engagement in AMS.	53-56
GP-pharmacist partnership based delayed antimicrobial prescribing model	GP and pharmacist are enthusiastic about partnership based delayed antibiotic use strategy to improve patient awareness.	60,
GP-pharmacist routine antimicrobial review model	There is a benefit of doing face-to-face 'case conferences to effectively do medication reviews, but case conferences are generally limited to complex patient cases. Though CPs role of antimicrobial prescription review has been supported by literature [11, 32-34, 61], there is a	11, 32-34, 61

	potential evidence gaps in relation to the elements of antimicrobial review processes, the mechanism of implementation of this model and assessment of the contextual feasibility.	
GP-pharmacist diagnostic stewardship model	Rapid diagnostic tests have been evolving to optimise antimicrobial therapy [66]. AMS diagnostics (e.g., point-of-care tests) translate test results faster for appropriate clinical action towards antimicrobial therapy [77]. Having these diagnostic tools in general practice and community pharmacy can be a source of collaboration between GPs and CPs. These tools will harness the processes of appropriate patient referrals, selection of antimicrobial and where delayed antimicrobial prescription might have undesired consequences for patient outcomes [68]. The evidence of effectiveness strongly supports that using these tests can substantially reduce antimicrobial use in primary care, but contextual feasibility and cost-effectiveness are not sufficient for policy actions in Australian primary care [66, 69-71].<br><br>In contrast, the state-wide use of point-of-care tests in routine pharmacy practices is increasing in the United Kingdom and the United States [7,14, 69-71]. The use of point-of-care tests service in the US community pharmacies was effective in reducing antimicrobial use in pharyngitis and influenza management. The model was feasible and acceptable to patients when a local GP-pharmacy collaborative practice models supported the patient referral system [7,14,71].	7,14, 66, 68, 69-71, 77

Supplementary Table S2: GUIDED checklist – a guideline for reporting intervention development studies

Item description	Pages
1. Report the context for which the intervention was developed.	3
2. Report the purpose of the intervention development process.	13-14
3. Report the target population for the intervention development process.	14
4. Report how any published intervention development approach contributed to the development process	14
5. Report how evidence from different sources informed the intervention development process.	13-14 and Table 1
6. Report how/if published theory informed the intervention development process.	5
7. Report any use of components from an existing intervention in the current intervention development process.	5
8. Report any guiding principles, people or factors that were prioritised when making decisions during the intervention development process.	14
10. Report how the intervention changed in content and format from the start of the intervention development process.	5-13
11. Report any changes to interventions required or likely to be required for subgroups.	14
12. Report important uncertainties at the end of the intervention development process.	12
13. Follow TIDieR guidance when describing the developed intervention.	13
14. Report the intervention development process in an open access format.	Table 1 and 2 and Saha et al [77]

[Reproduced from Duncan *et al.* 2020. Duncan E, O'Cathain A, Rousseau N, Croot L, Sworn K, Turner KM, Yardley L, Hoddinott P. Guidance for reporting intervention development studies in health research (GUIDED): an evidence-based consensus study. BMJ open. 2020;10(4): e033516.]
